# Novel approach to study the perception of animacy in dogs

**DOI:** 10.1371/journal.pone.0177010

**Published:** 2017-05-04

**Authors:** Judit Abdai, Cristina Baño Terencio, Ádám Miklósi

**Affiliations:** 1 Department of Ethology, Eötvös Loránd University, Budapest, Hungary; 2 University of Valencia, Valencia, Spain; 3 MTA-ELTE Comparative Ethology Research Group, Budapest, Hungary; University of New England, Australia, AUSTRALIA

## Abstract

Humans tend to perceive inanimate objects as animate based on simple motion cues. So far this perceptual bias has been studied mostly in humans by utilizing two-dimensional video and interactive displays. Considering its importance for survival, the perception of animacy is probably also widespread among animals, however two-dimensional displays are not necessarily the best approach to study the phenomenon in non-human species. Here we applied a novel method to study whether dogs recognize a dependent (chasing-like) movement pattern performed by inanimate agents in live demonstration. We found that dogs showed more interest toward the agents that demonstrated the chasing-like motion, compared to those that were involved in the independent movement. We suggest that dogs spontaneously recognized the chasing-like pattern and thus they may have considered the interacting partners as animate agents. This methodological approach may be useful to test perceptual animacy in other non-human species.

## Introduction

Attribution of animacy or psychological traits to inanimate agents seems to be a general phenomenon in humans starting early during ontogeny (e.g. [1–5]). Tremoulet & Feldman [6] showed that even a single moving geometric figure can be identified by adult observers as animate based on simple motion cues. Recent research found that newborn human infants are already sensitive to self-propelledness [5], and newly hatched, naïve domestic chicks (*Gallus gallus domesticus*) also prefer self-propelled geometric figures to those moving with constant speed or their motion is the result of physical contact [7,8]. Thus it seems that there are inborn predispositions to these cues that may be widespread in vertebrates apart from the fact that basic motion cues of animacy are sufficient to trigger the perception of animacy [5,7,9] (but see [10,11]).

Spatiotemporal contingencies among moving inanimate agents can trigger the impression of interactive events, such as chasing (e.g. [2,3,12,13,14]). Considering that the quick recognition of animate entities can be important for survival, the over-attribution of such property seems to be advantageous and it is probably widespread in the animal kingdom (e.g. [15]). We should also consider that chasing may occur during predatory as well as social interactions. However, only limited amount of studies investigated this phenomenon in non-human species (e.g. [7,16,17]).

Objects displaying self-propelled motion are described as animate, while agency is attributed when goal-directed behaviour is observed (e.g. [8,18,19]). However, the two concepts cannot be fully separated, as animacy can be perceived without goal-directed behaviour, but attributing agency without animacy is not possible (e.g. [14]). Chasing contains both information because it involves, for example self-propelled motion and violation of Newtonian mechanics, and the motion can be described as goal-directed, while non-mechanical contingency is also a general characteristic of it.

In studies conducted with human infants and adults, researchers used two-dimensional video and interactive displays that relied on the visual perception of participants. They used chasing-like motion as a dependent movement pattern performed by inanimate agents (geometric figures) that could lead to the spontaneous recognition of a social event based simply on motion information (e.g. [2,13,14,20]). Rochat and colleagues [2,3] presented the side-by-side video display of chasing and independent motion patterns to infants and adults, and measured looking duration (preference) toward the stimuli. Frankenhuis et al. [20] used a similar setup to test whether infants’ preference to a chasing pattern is due to its configuration or some features of it (acceleration, high turning rates, and attraction) are responsible for the preference.

The measure of looking duration at stimuli is a widely used procedure but it provides only an indirect access to the underlying mental processes and how it reflects behaviour function in real situations. Another widely used method to investigate whether participants detect a chasing-like pattern in such displays is by asking them directly about their thoughts on the stimulus. For example, in the so called *Find-the-Chase* task adults watched a video display of moving geometric shapes and they had to report whether they perceived chasing in the display (e.g. [13,21,22]). In some cases this method is combined with the measurement of subjects’ looking pattern toward the stimuli (e.g. [2]).

In most studies researchers relied on the looking patterns of participants or on adults’ reports and ratings about the stimuli when arguing for the percept of animacy (e.g. [1,2,6,21,22]). However, Scholl and Gao [23] claim that these methods do not allow a clear separation of perception and cognition as the underlying process. In a series of studies Gao et al. (e.g. [13,14,24]) used the *Don’t-Get-Caught*! task to test whether participants can pinpoint the chaser among many moving agents with the same physical properties. In this task, participants had to avoid the chaser by controlling one of the figures on the screen and they could only rely on the motion characteristics of the chaser to succeed [13,14]. Authors proposed that the *Don’t-Get-Caught*! task serves as a better approach to study visual perception in relation to animacy detection, because it measures participants’ perception more objectively and we can also obtain information about the effect of the percept on subjects’ behaviour [14].

Only a few studies have investigated perceptual animacy in non-human species to date [7,8,16,17]. For example, researchers used two-dimensional displays applying the *go/no-go discrimination* task (which requires initial training). Neither in pigeons (*Columba livia*) [16], nor in squirrel monkeys (*Saimiri sciureus*) [17] authors could provide convincing evidence on whether subjects perceived the specific interaction among the agents, a dependent motion pattern. However, we suggest that the use of training when investigating perception makes it difficult to separate whether the observed behaviour of subjects is due to the perception of the specific features of the motion or they used another strategy related to their initial training. A better approach has been applied with newly hatched chicks [7,8], in which researchers tested subjects’ preferential approach to one of two stimuli.

The above procedures have several advantages, for example, in case of video displays the stimulus can be manipulated more strictly, and the experimental conditions are better controlled. However, we argue that these methods either do not provide convincing evidence on the attribution of animacy to inanimate agents on the level of perception, or they are difficult to implement in comparative investigations (but see [7,8]).

Here we applied a novel approach to study the perception of animacy in non-human species, specifically in dogs (*Canis familiaris*). We presented dogs a dependent and independent motion pattern in live demonstration by using moving inanimate agents (UMO–Unidentified Moving Object). UMOs have been used in previous studies as interactive partners to investigate social behaviour of dogs (e.g. [25,26]); authors found that dogs tend to show social (or social-like) behaviour toward these agents following a short interaction.

Dogs observed two UMOs demonstrating a dependent motion pattern that resembled a chasing interaction (hereafter chasing) and in a separate trial they observed two UMOs moving independently from each other (all subjects observed both demonstrations after each other). After this phase, dogs were presented with two UMOs (one from each trial). Dogs were allowed to behave freely for one minute in the presence of the UMOs that stood still and did not react to subjects’ behaviour. We hypothesized that dogs show more interest in the UMOs that demonstrate the chasing pattern because they recognize the specific interaction based on the motion characteristics of the UMOs. The recognition of the dependent motion pattern between the agents facilitates the attribution of animacy to them, which shifts subjects’ attention toward these UMOs compared to those that moved independently (more mechanistic agents).

## Materials and methods

### Ethics statement

Ethical approval was obtained by the National Animal Experimentation Ethics Committee (PE/EA/2484-4/2016). Owners provided a written consent form to voluntarily permit their dogs to participate in the study.

### Subjects

We tested 35 dogs that have not had previous experience with UMOs. We excluded 11 dogs: *one* dog due to technical difficulties with one of the UMOs during the Observation phase; *two* dogs, because they showed distress in the presence of the UMOs; *one* dog was influenced by the owner during the Test phase; *one* dog looked one of the demonstrations less, than 20% of the length of the demonstration; *three* dogs, because they did not approach any of the UMOs during the test phase; and *three* dogs due to procedural problem (either the UMOs crashed into each other, or they crashed into the obstacles more than two times by moving them at least 0.5 m away from their original locations; both of these could be salient cues affecting dogs’ behaviour toward the agents later). The remaining 24 dogs were from different breeds (15 females; mean age (year)±SD 4.96±2.99).

### Apparatus

**Fig 1:**
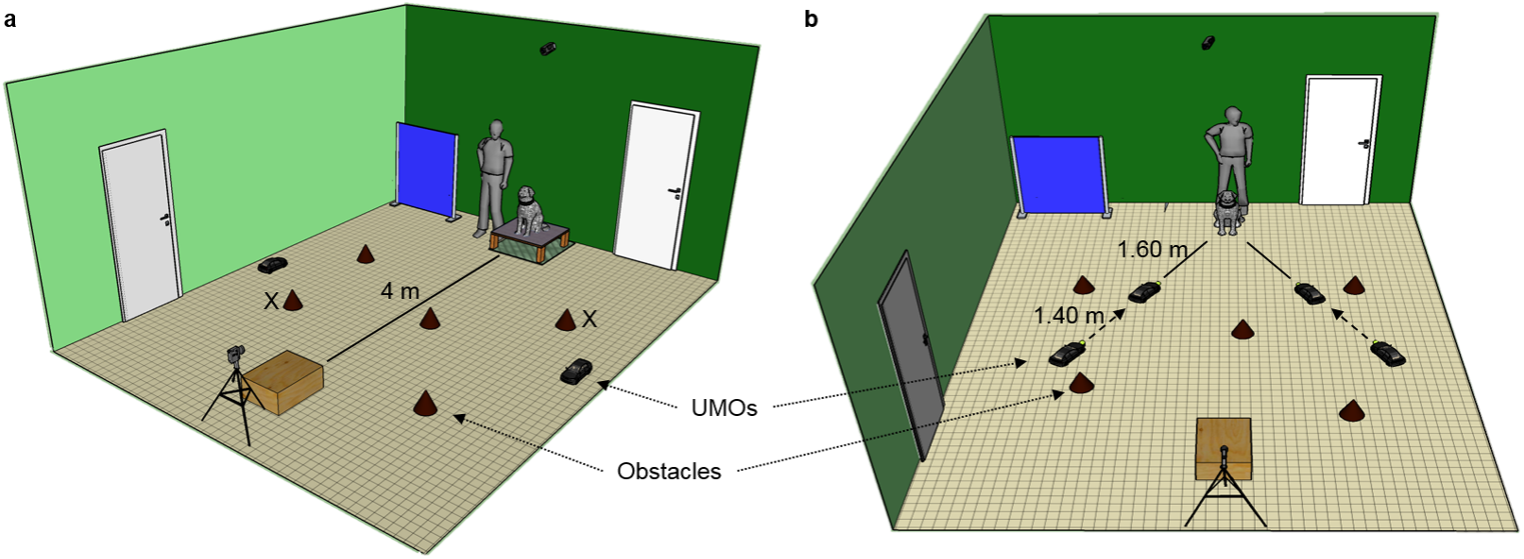
**Experimental set up in the (a) Observation phase (b) Test phase.** Dashed arrows indicate the movement of the UMOs toward the dog; X marks indicate the starting points of the chasing and independent patterns.

Dogs were tested in a 6.27 m x 5.40 m test room at the Department of Ethology, Eötvös Loránd University (for the full experimental set up see Fig 1). Five identical brown pots were placed in the room upside-down serving as obstacles during the demonstration of the movement patterns. During the observation dogs were sitting on a wooden platform covered with artificial grass (H x W x L: 25 cm x 80 cm x 80 cm), in the middle next to one of the walls. The platform was on a green PVC mat to make it more stable and prevent the slipping. The platform helped dogs’ view to the room and separated them physically from the interacting UMOs during the observation phase. The first seven dogs were tested with a smaller and less stable platform (25 cm x 55 cm x 55 cm). We compared the looking duration during the demonstrations between the seven dogs tested with the smaller platform and the first seven dogs that have been tested with the larger platform. Based on the data analysis the differences between the platforms had no effect on dogs’ behaviour (Independent samples Mann-Whitney U test: Looking at the chasing demonstration, U = 24, p = 1.000; Looking at the independent demonstration, U = 19, p = 0.535); thus we included these dogs into the final analysis as well.

In front of the dog, next to the opposite wall, we placed a wooden box (38 cm x 48 cm x 80 cm) with a front opening (20 cm x 20 cm) facing to the dog. In the corner on the right side of the dog there was a blue cartonplast occluder (1.27 m x 5.40 m) that was used to cover dogs’ view to the room when required.

In the test phase we used two tennis balls that we put on two plastic plates (8 cm × 8 cm) which had metal sheets on their sides. All embodiments of the UMOs were equipped with magnets on their fronts. The plates (containing the balls) could be moved by attaching them to the magnets by the metal sheets.

Tests were recorded by three cameras: one hand camera (Sanyo Xacti) mounted on a tripod behind the box oriented toward the dog, and two fish-eye optic cameras (Mobius ActionCam) synchronised with each other hanging from the ceiling above the dog and the wooden box.

### The inanimate agents (UMOs)

**Fig 2:**
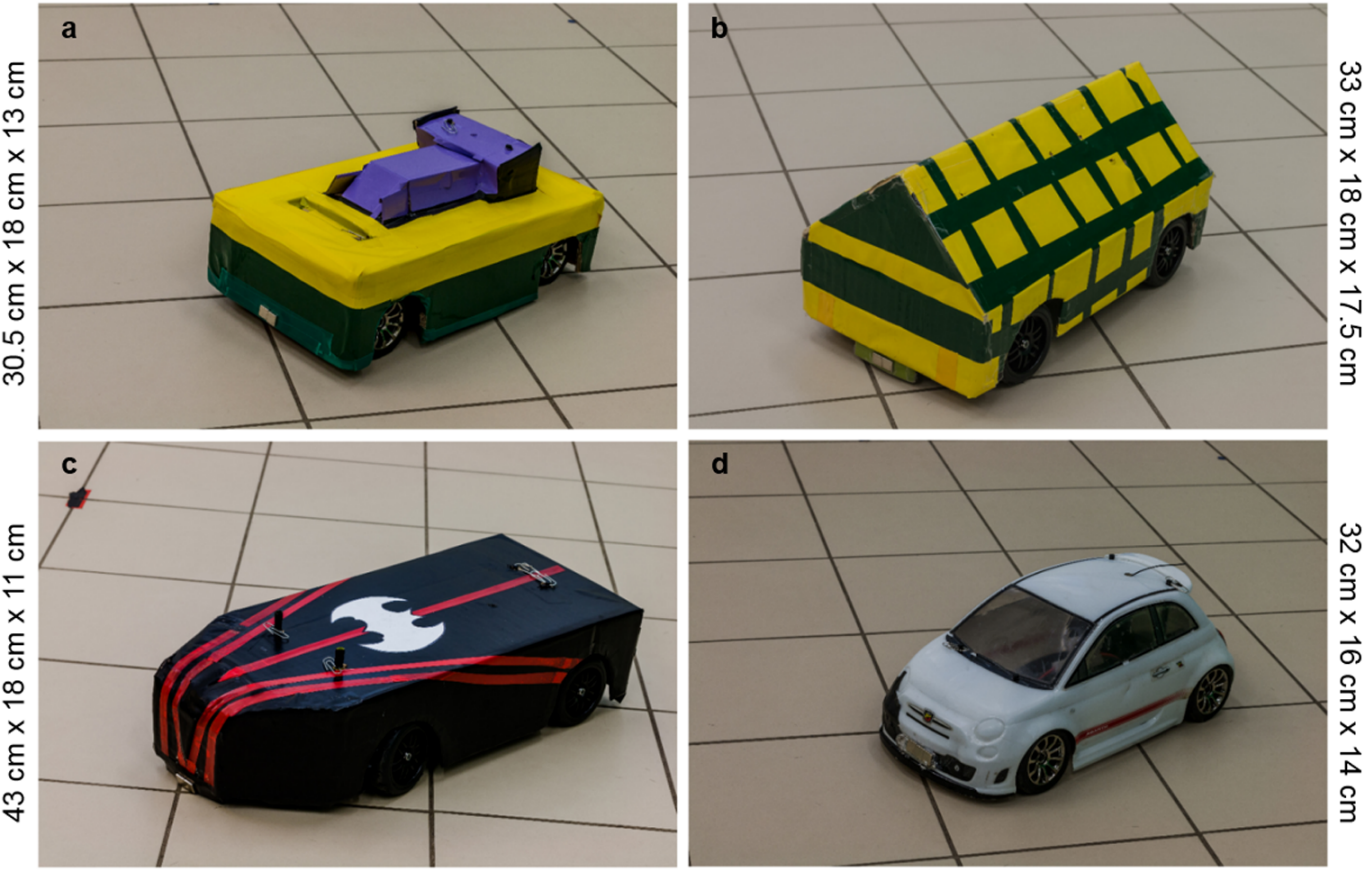
Embodiments of the UMOs. The (a) and (d) are the embodiments of UMO 1; and (b) and (c) are the embodiments of UMO 2. We used (b) and (d) for the independent demonstration, and (a) and (c) for the chasing demonstration. In the test phase we had two pairs: (a) and (b); (c) and (d).

Two remote-controlled cars were used as UMOs (#32710 RTR Switch Abarth 500, 28 cm x 16 cm x 13 cm; and #7304 Traxxas Ford Mustang Boss 302; 31 cm x 18 cm x 11.5 cm). The UMOs were controlled by Experimenter (E) 1 and 2 from outside through the fish-eye optic cameras. Both UMOs had two different embodiments in colour and shape (one for the demonstration of the dependent pattern and one for demonstrating the independent motion pattern; Fig 2). In the following we refer to the remote-controlled cars as UMO 1 and UMO 2 based on the body of the car itself (independently from the embodiment).

### Design of the movement patterns

In the design of the chasing pattern we took into consideration motion characteristics that have been found as important to recognize a chasing pattern in former studies (e.g. [13,14,20]): (1) The chaser UMO moved directly toward the chased UMO following the shortest route (e.g. if the chased UMO went around an obstacle, the chaser UMO cut the turn and moved directly toward the chased UMO); (2) The chaser UMO oriented toward the chased UMO; (3) The chased UMO executed frequent directional changes during escape; (4) Both UMOs displayed sudden changes in their speed (e.g. when the chaser UMO came within ca. 1 m the chased UMO accelerated).

In case of the independent pattern, both UMOs followed different routes and moved independently from each other. For the design of these routes we gave numbers to the five obstacles and generated a random sequence with numbers from 1 to 5 (CarChase program developed by Bence Ferdinandy). This sequence gave the order of the obstacles that should be passed by (the starting points of the demonstrations were fixed; see the Procedure). For the two UMOs that participated in the independent demonstration we generated separate sequences, the number of elements of the sequences was given based on the chasing pattern (how many obstacles the UMOs passed by during this). The program generated the sequence in a way that the UMOs spend equal time in the two parts of the room to avoid asymmetrical cues. At some points of their routes (depending on certain constraints, such as the avoidance of sudden collision) the UMOs increased and decreased their speed, they crossed each other’s path, and several times followed the same route right after each other (with a larger distance as in case of the chasing), in order to reduce the difference between the chasing and the independent demonstrations (we aimed to counterbalance the number of these events between the demonstrations). There was no difference in the number of accelerations and sharp direction changes between the two demonstrations (Paired-samples t test: *Acceleration*, t_23_ = -1.457, p = 0.159; *Direction change*, t_23_ = 1.748, p = 0.094; see Table 1).

**Table 1:** Quantitative information on the movement of the UMOs in the chasing and independent demonstrations.

	Chasing	Independent
**Average number of accelerations (± SD)**	19.83 (± 3.41)	19.21 (± 3.68)
**Average number of sharp direction changes (ca. >110°) (± SD)**	11.5 (± 2.90)	11.96 ± (2.69)

Due to technical reasons we used only one predetermined route for the chasing, and one for the independent movement pattern. In another study we have found that dogs discriminate between a chasing and an independent movement pattern performed by geometric shapes, when using video displays (Abdai et al., under review). Taking into consideration that in that study we used different chasing patterns (15 different chasing patterns generated with the same parameters) and did not find any influence of the displayed route itself, we suggest that probably it was not necessary to use different routes in case of the different subjects in the present study. The experimenters controlled the same UMOs in all the phases and tests (E1 –UMO 1 and E2 –UMO 2). The roles of the UMOs were not interchangeable, UMO 1 was always the chaser and UMO 2 the chasee in the chasing pattern; UMO 1 was going inside the box during the independent pattern and UMO 2 in the chasing (see the Procedure).

### Procedure

#### Observation phase

After the owner and the dog entered the room, the dog explored the room while E1 informed the owner about the procedure. The dog sat on the platform orienting toward the box, the owner stood/sat next to the dog while holding it on a leash. E1 put the occluder in front of the dog while E2 placed the UMOs next to the middle of the walls, on the sides of the dog (Fig 1A). In case of the chasing demonstration UMO 2, in case of the independent demonstration UMO 1 was on the left side. E1 removed the occluder and the experimenters left the room. Throughout the test the experimenters used the door next to the dog, the owner with the dog the other door (Fig 1).

Both patterns started with a familiarization during which the UMOs moved simultaneously around the room (elliptical route). After 1.5 rounds the UMOs started to follow their predetermined route by turning away from the elliptical routes at the starting points which were the same in case of the chasing and independent demonstrations (see Fig 1). Both observation trials ended with one of the UMOs going inside the box (see above) (mean time of the demonstration (s)±SD, chasing: 71.06±8.21; independent: 94.76±4.24; the longer duration in case of the independent motion trial occurred probably because if the UMOs were too close to each other when UMO 1 went into the box, UMO 2 made an extra round to avoid any motion cues that could have been perceived as an interaction among them). After the first observation trial ended, the dog and the owner left the room. The dog was not allowed to go to the UMOs. E1 and E2 changed the embodiments of the UMOs. The owner and the dog re-entered the room, they went back to their original location and the test was continued with the second observation trial. At the end of this trial the owner and the dog left the room again.

All subjects observed both the chasing and independent patterns. We counterbalanced for the order of the movement patterns between subjects.

#### Test phase

Before the owner and the dog came back, E1 and E2 put away the platform. They changed the embodiment of one of the UMOs, thus one of the UMOs had the embodiment from the independent, while the other had the embodiment from the chasing demonstration. Due to the different sizes of the cars we could only use two combinations (pairs) of the embodiments (Fig 2). The used pairs were counterbalanced between subjects.

The owner hold the dog at its original position (without the platform) and E1 put the occluder in front of the dog. E2 placed the UMOs to their predetermined location, 3–3 m away from the dog (Fig 1B). E2 brought in two balls and two small plates and stood in front of the dog, in a 1.5 m distance to the middle. E1 removed the occluder and took one of the balls and plates from E2 invisibly to the dog. E1 and E2 stood next to each other, hiding the balls and plates behind their backs. E1 and E2 simultaneously called the dog’s attention by saying its name and showed the balls to the dog while putting them on the plates. They attached the plates to the UMOs. If the dog focused only on one of the UMOs, the experimenters gave a non-social signal to the dog (both of them knocked on the floor with their hands in front of the UMOs without eye-contact). E1 and E2 showed their empty hands to the dog and left the room. The UMOs moved closer to the dog and stopped 1.6 m away from it. After 5 s as the UMOs stopped, the owner released the dog that could behave freely (approach and touch the UMOs and the balls attached to them) for 1 min. The UMOs stood still and did not react to the dogs’ behaviour. Due to technical problems the duration of the Test phase was less than 1 min in the case of six subjects (mean time (s)±SD 56.44±8.04). We did not exclude these dogs, but we used the time from the moment the dog was released until the end as maximum time of the Test phase for the analysis of the latency data (see Behavioural variables and data analysis).

We counterbalanced between subjects the sides of the UMOs. For half of the subjects E1, for the other half of the subjects E2 attached the ball to the UMO that demonstrated the chasing pattern.

### Behavioural variables and data analysis

Tests were analysed with the Solomon Coder 15.11.19 (by András Péter: http://solomoncoder.com).

#### Observation phase

Looking duration toward the chasing or independent demonstrations (%): the looking duration toward the UMOs (s) during the chasing or independent demonstrations (from the UMOs first deviation from the elliptical route until both UMOs stopped) / total time of the given demonstration (s) * 100.

#### Test phase

First approach (0/1): the UMO that the dog approached first. First touch (0/1): the ball attached to the UMO that the dog touched first. We scored each trial as 0 if the dog approached/touched first the UMO (the ball attached to it) from the independent demonstration and as 1 if the dog approached/touched first the UMO (the ball attached to it) from the chasing demonstration.

Latency of first approaches (s): latency of approaching the UMOs after the owner let the dog go. Latency of first touches (s): latency of touching the balls attached to the UMOs after the owner let the dog go. Latency of first grabs (s): latency of grabbing the balls attached to the UMOs after the owner let the dog go.

For the statistical analysis we used IBM SPSS Statistics 22 and R software version 3.2.4 (R Development Core Team (2008)).

We compared dogs’ looking duration toward the chasing and independent demonstrations during the Observation phase with related-samples Wilcoxon signed rank test (IBM SPSS). By one-sample binomial test (0.5 chance level) we analysed whether dogs approached and touched first the UMOs (the ball attached to them) from the chasing demonstration (IBM SPSS). We used a mixed-effects Cox regression (R coxme package) to compare the differences between the latencies of the first approaches, first touches and first grabs of the UMOs (the balls attached to them). We used Cox regression to test whether dogs’ behaviour was influenced of whether the chaser or the chased UMO was in the room in the Test phase (latency of approach, touch and grab) (IBM SPSS).

We used Generalized Linear Models (GzLMs) to analyse whether the movement of the UMOs (started to move first, stopped later), or their different distances from the dog in the Test phase had an effect on dogs’ first approach of the UMOs.

Inter-observer reliability for the looking duration (%) variable was tested on a random subsample of the recordings (20% of the subjects) by an independent observer (NC) (Cronbach’s alpha was 0.713).

## Results

Related-samples Wilcoxon signed-rank test showed that dogs’ looked at both demonstrations for equally long durations during the Observation phase (Looking at the chasing demonstration: mean (s)±SD 76.122±20.623; Looking at the independent demonstration: mean (s)±SD 73.102±13.618; Related-samples Wilcoxon signed-rank test: N = 24, z = -1.257, p = 0.209).

**Fig 3:**
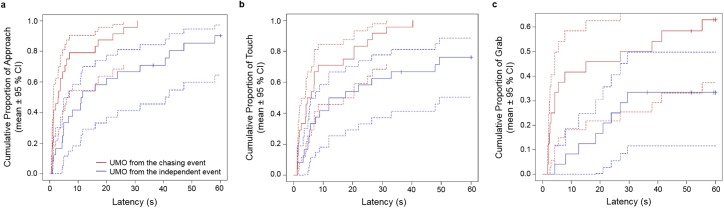
**Latencies of (a) first approach of the UMOs, (b) first touches and (c) first grabs of the balls attached to the UMOs.** The figures show the cumulative proportion of individuals presenting the given behaviour at a given time (Mixed effects Cox regression).

With mixed effects Cox regression, we found significant difference in the latency of first approach of the UMOs, and the latency of first touch and first grab of the balls attached to the UMOs. Dogs approached the UMOs from the chasing demonstration with approximately 4.4 times higher chance within a given time, than the UMOs from the demonstration of the independent motion (Log-likelihood comparison: χ2 = 14.475; p<0.001; exp(β)[95% CI] = 4.359[2.11; 9.02]; p<0.001) (Fig 3A). Also, dogs touched with approximately 4.3 times higher chance within a given time the ball attached to the UMOs from the chasing demonstration, than the ball attached to the UMOs from the demonstration of the independent motion (Log-likelihood comparison: χ2 = 13.619; p = 0.001; exp(β)[95% CI] = 4.26[2.02; 8.99]; p<0.001) (Fig 3B). We also found that dogs grabbed the ball from the UMOs that demonstrated the chasing pattern with approximately 6.3 times higher chance within a given time, than from the UMOs that demonstrated the independent movement (Log-likelihood comparison: χ2 = 10.509; p<0.001; exp(β)[95% CI] = 6.325[2.21; 18.13]; p<0.001) (Fig 3C).

**Fig 4:**
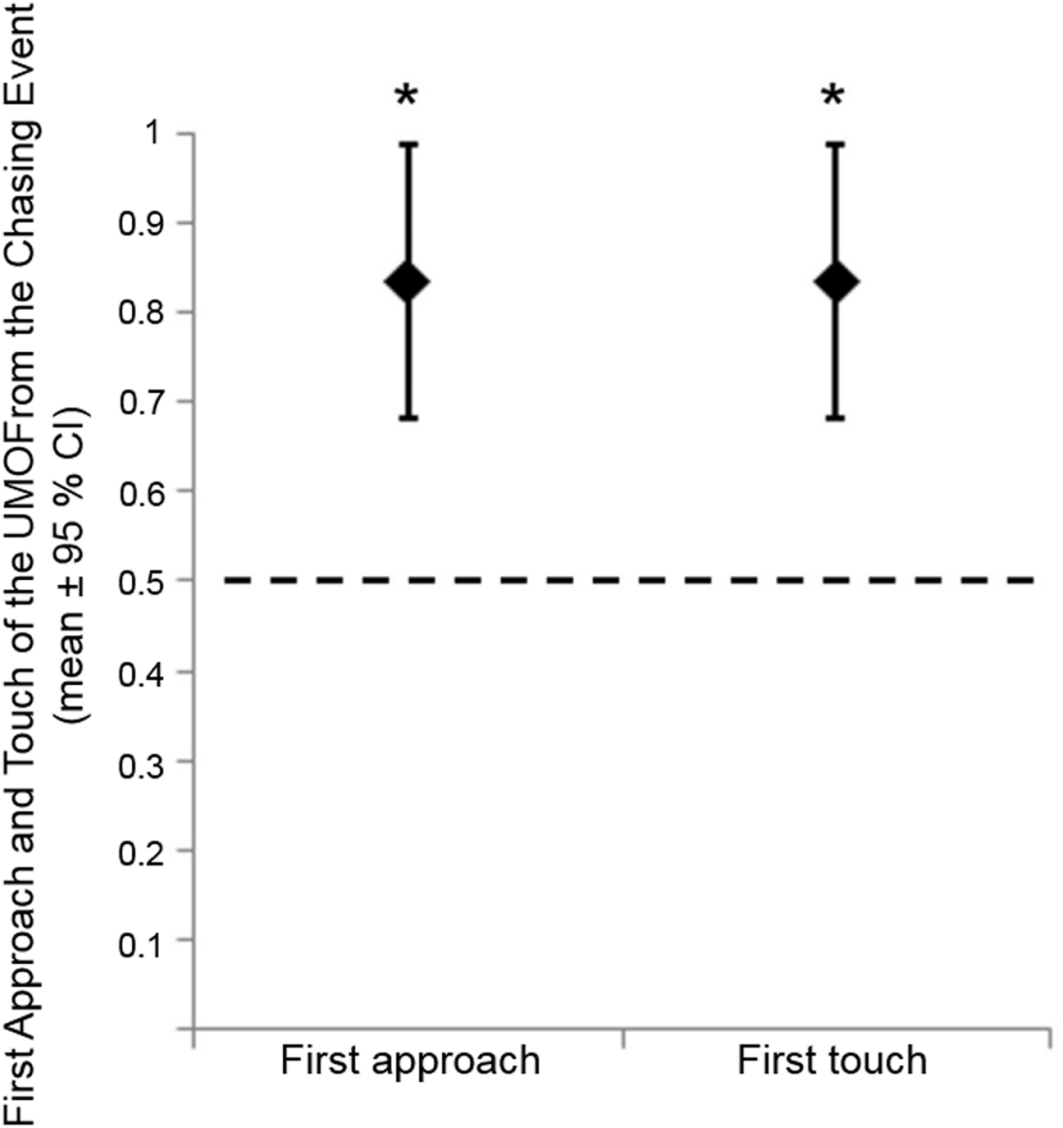
Dogs’ first approach and first touch of the UMOs in the Test phase. The dashed line represents the 0.5 chance level. The value 0 represents the UMOs from the independent demonstration and the value 1 the UMOs from the chasing demonstration (* p<0.01).

Based on the results of the one-sample binomial test (0.5 chance level), dogs approached and touched the balls attached to the UMOs from the chasing demonstration significantly more often for the first time (approach: p = 0.002; touch: p = 0.002; Fig 4).

Dogs’ behaviour was independent of whether the chaser or the chased UMO was in the room in the Test phase (Cox regression–approach: exp(β) = 1.521, p = 0.331; touch: exp(β) = 1.776, p = 0.183; grab: exp(β) = 2.104, p = 0.161).

We found that dogs’ first approach of the UMOs were not influenced by which of the UMOs started to move first and stopped later when they moved closer to the dog in the Test phase. The difference in their distances from the dog also did not have an effect (GzLM: Moved first, χ2(2) = 0.863, p = 0.650; Stopped later, χ2(2) = 9.15x10^-7^, p = 1.000; Closer, χ2(2) = 0.187, p = 0.911).

## Discussion

It seems that overall dogs were more interested in the UMOs from the chasing demonstration; they approached these UMOs sooner, and also touched and took away the balls attached to these agents earlier. We propose that dogs’ behaviour reflect the recognition of the chasing pattern, and as a result they considered these UMOs as potential interactive partners. It is also a plausible explanation that dogs showed such preference, because during their daily lives both the balls and the chasing pattern can be associated with playing. This explanation also supports the recognition of the dependent motion pattern as chasing.

The novel method applied here can be a useful approach to study the spontaneous recognition of animacy for several reasons. Considering that subjects behave freely during the Test phase, they can engage in direct interaction with the agents (although these do not react to the dogs’ behaviour), which allows the assessment of a wider range of behaviour, including preference of one or the other UMO. We propose that this method offers a more direct measure of the perception of animacy because subjects have the chance to initiate an interaction. Also, in former studies that examined the duration of looking at the stimuli (e.g. [2], Abdai et al., under review), the recognition of the dependent motion pattern was claimed indirectly (the recognition of the dependent pattern was claimed based on the longer looking toward the independent pattern). However, in the present study the conclusions were drawn from direct measurement of subjects’ behaviour, from their preference toward the agents that demonstrated the chasing pattern (see also [7,8]). We suggest that the present data provide stronger evidence on the presence of the phenomenon. Our new method serves better the call from other researchers who suggested that by studying participants’ implicit interactive behaviour, more objective data can be obtained, thus it may be a more suitable way to test the perception of animacy [14,24,27]. In line with this, here we also investigated whether the observation of the dependent motion pattern performed by inanimate agents would have an effect on subjects’ behaviour. In addition, our new method does not require initial training, thus subjects do not habituate to the demonstrated patterns which could have an impact on their behaviour.

Subjects’ preference between two stimuli is often measured by the duration of subjects’ look at the stimuli. We found that dogs’ looking duration did not differ between the demonstrations of the movement patterns, thus it can be raised that they did not prefer any of them. However, we suggest that differences/similarities in the looking time toward stimuli are most reliable when the stimuli are displayed simultaneously, and as a result subjects need to divide their attention between them. In that case we would expect that they spend more time looking at the stimulus they are more interested in. In the present study we showed only one motion pattern at a time to dogs, thus there was no cost of looking at the less interesting pattern. We suggest that dogs’ similar looking duration toward the dependent and independent motion patterns does not indicate the lack of preference or recognition in this specific case.

Similarly as in former studies (e.g. [2,13,14]), we referred to the dependent pattern as chasing and we used motion characteristics for the design of the movement that have been discussed in relation to this specific interaction (e.g. [13,14]). Although we cannot conclude that the displayed pattern was recognized specifically as chasing by our subjects, we suggest that the different behaviour of our participants that was shown toward the UMOs could be only due to the difference in their motion patterns (dependent vs. independent).

Frankenhuis et al. [20] found that acceleration and reduced distance among objects (animacy-related cues) explain infants’ preference for chasing patterns compared to independent motion patterns. Considering that here we counterbalanced for many aspects of the motion (e.g. number of acceleration and direction changes) between the dependent and independent motion demonstrations, one can argue that our results might serve as evidence for agency attribution. However, Tremoulet & Feldman [28] suggested that the attribution of animacy requires the attribution of some mental capacity to the agent as well. As we do not know exactly which characteristic(s) influenced dogs’ behaviour, we can only conclude that their preference might be due to the recognition of animacy (as agency cannot be perceived without animacy, but the reverse is not true; see [14]).

The physical characteristics of the motion patterns are important in the perception of animacy (e.g. [13,14]). Although some studies have been aimed at investigating how the changes in some parameters of the motion characteristics influences this perception (e.g. [13,14,20]), still many details have remained unexplored. For example, we propose that the exact values of these parameters (e.g. average speed) may have an effect on the perception of animacy, meaning that the given motion pattern may give rise to the percept of animacy only within a specific range of the values of the parameters (see also [13,14]). The novel method presented here can facilitate research aimed at investigating such details of the perception of animacy. We further suggest that there may be ecologically relevant differences among species in relation to the exact motion characteristics that can lead to the recognition of inanimate agents as animate entities. By the means of this method a wide range of species could be tested, even in their natural habitat. This would allow us to assess potential differences in the criteria of attributing animacy to inanimate agents in species with different life style, for example, social vs. non-social species, herbivores vs. predators.

Future investigations conducted on different species are required to draw conclusions about the evolutionary background of the perception of animacy. We propose that the methodological approach we introduced in the present study can be used more widely among species to test this phenomenon, which would allow more direct comparison of their perceptual abilities. Nowadays more and more robots are available, in cases of which many parameters of the movement (e.g. speed) can be set previously or the routes can be drawn beforehand. We believe that technological advances enable the refinement of our method to be able to control better for many parameters of the motion in the future.

## Supporting information

S1 Dataset Measurement data of subjects. (XLSX)

S1 Video Video demonstration of the procedure. (MP4)
